# 24-month randomized controlled clinical trial assessment of surface texture, color stability, and marginal discoloration of sonic activated bulk-fill resin composite according to USPHS and FDI criteria

**DOI:** 10.1186/s12903-025-06611-0

**Published:** 2025-07-26

**Authors:** Ashraf Aref, Safaa Abd-Elhakim, Mona Riad

**Affiliations:** 1 Conservative Dentistry Department, Faculty of Dentistry, Minia University, Minia, Egypt; 2 Conservative Dentistry Department, Faculty of Dentistry, Cairo University, Cairo, Egypt

**Keywords:** Bulk-Fill resin composite, Sonicfill, Clinical evaluation, USPHS criteria, FDI criteria, Clinical trial

## Abstract

**Background:**

To assess the surface texture, color stability, and marginal discoloration of sonic-activated bulk-fill resin composite (BFRC) in comparison to non-sonic-activated BFRC, over 24 months, by two different evaluation criteria.

**Methods:**

30 adult patients, each presented with at least two carious lesions, either Class I or Class II cavities in their posterior teeth, were selected and subdivided randomly into two identical groups: one group restored with *Sonicfill 3* and the other group restored with the *X-tra fill*, with total of 60 restorations for both groups. Surface texture, color stability, and marginal discoloration were assessed at baseline (1 week), then at 3, 6, 12, and 24-month periods, applying the modified United States Public Health Services (*USPHS*) criteria and World Dental Federation (*FDI*) criteria.

**Results:**

After the 24-month follow-up evaluation period, there was a non-significant difference in the clinical outcomes between both tested groups, with a non-statistical difference between the results’ outcomes evaluated by USPHS or FDI criteria, where all restorations were considered clinically successful by both criteria.

**Conclusions:**

The surface texture, color stability, and marginal discoloration of both tested BFRCs over 24 months were considered clinically successful, either with or without the use of sonic energy. Regarding both criteria, they were reliable, comparable, and suitable for evaluating the clinical performance of RC restorations.

**Trial registration:**

The current study underwent registration at www.clinicaltrials.gov and obtained the unique identification number NCT04926883 for its protocol on 03/06/2021.

## Background

The clinical application of resin composite (RC) materials as dental restorations is steadily increasing. Adhesive restorative materials fulfill the requirements of minimally invasive dentistry and enable clinicians to preserve intact dental tissues. There is substantial clinical evidence supporting the success of direct and light-polymerized RC restorations in posterior teeth [1]. Among their advantages, they may account for the affordability, longevity, and remarkable aesthetic and mechanical properties provided by various components, in order to meet patients’ increasing aesthetic expectations even in posterior teeth [2, 3].

Manufacturers have been working to improve the physical and optical characteristics of RCs ever since they were first introduced to mimic natural-looking teeth and make handling easier [4]. Despite these favorable improvements, polymerization shrinkage remains a significant challenge. Although there are many ideas for packing RC materials in cavities to avoid polymerization depth limitations and overcome polymerization shrinkage stress, placing them with a layering technique of 2 mm thickness or less is recommended. This technique has become a frequently used method in clinical practice [1, 5, 6]. However, deep cavity restoration with incremental technique requires time and effort, and postoperative sensitivity may still occur [7].

Because of its improved translucency, which allows for a deeper cure, the bulk-fill resin composite (BFRC) was created in response to the increasing need for more efficiency packing in a single increment of 4 to 5 mm thickness. Shorter curing times and greater depth of cure can result from optimizing the light curing composite’s photo-initiator system, reducing the number of composite layers and limiting polymerization shrinkage [7, 8]. However, sonicfill RC (Kerr, Orange, CA, USA) was introduced as the only known sonically activated composite by its special air-driven handpiece, which decreases its viscosity and in turn allows building posterior restorations up to 5 mm thick in one step, decreasing the workflow and operation time at the chairside. Once the sonication is terminated, the viscosity of the material increases. Although sonicfill is less translucent than other conventional BFRCs, the depth of polymerization does not depend only on the translucency of the material after photo-polymerization [9].

One of the significant drawbacks of RCs is their potential to change color over time as a result of intrinsic and extrinsic factors. For this reason, the shade selection and color stability of restorative materials have become crucial in deciding whether restoration will be maintained for a long time or needs replacement due to patients’ increasing demands for aesthetics [4].

To standardize the assessment of operative techniques or restorative materials in clinical trials, various criteria have been proposed [4]. Nonetheless, establishing a protocol to standardize the assessment of clinical performance and success of different dental restorations by professionals is a primary objective to achieve predictable and standardized dental care. The Ryge criteria or the United States Public Health Service (USPHS) criteria and its modification, in addition to the World Dental Federation (FDI) criteria and its revised version, are among the criteria established for this purpose and play important roles in the assessment of dental restorations’ clinical performance and additional evaluations of the applicability and suitability of various dental restorations [10–16]. The assessment of the clinical acceptability of numerous restorations relies on the success rate of these restorations according to the criteria used. The US Public Health Service (USPHS) and FDI (World Dental Federation) guidelines are the most commonly used criteria for evaluating RC restorations [10–12, 15, 17–19]. Both criteria are based on the assessment of different parameters, such as aesthetic, functional, and biological criteria, and can be modified according to the user’s needs. Criteria such as color matching, marginal discoloration, surface structure, retention, marginal integrity, anatomic form, secondary caries, and postoperative sensitivity, which are clinically significant for dental restorations, have been developed for evaluation. The restoration’s defined properties according to the modified USPHS criteria are evaluated with Alpha, Bravo, Charlie, and Delta scores according to the assessment of the patient, visual examination, and radiographs. These scores are denoted as Alpha, indicating the best score, whereas Delta indicates the worst score [12].

Recently, FDI criteria have been introduced in literature as “standard criteria” that are more sensitive for identifying differences in dental restorations [14]. The FDI criteria were published in 2007 and classified into three main categories for evaluation of restorations: functional, biological, and aesthetic criteria, which were divided into sub-groups within each group. The subcategories’ scores, which range from 1 to 5, determine the final points for the three main categories. According to the evaluation of these criteria, the restoration is clinically sufficient if it receives scores of 1, 2, or 3, clinically insufficient but repairable if it receives a score of 4, and totally clinically insufficient if it receives a score of 5.

Multiple in vitro studies have tested sonic-activated BFRC, but fewer in vivo studies were available when this research was started. The novelty of this study was the ability to conduct a clinical assessment of the color properties of sonic- and non-sonic activated BFRC using the two most commonly used clinical evaluation criteria, USPHS and FDI. It also considered the controversies about their sensitivity to detect differences and providing a valuable comparative perspective on evaluation standards.

Therefore, this study aimed to compare the surface texture, color stability, and marginal discoloration of two types of BFRC (sonic- and non-sonic-activated) in posterior teeth over 2 years and to examine whether choosing an expensive, specially-equipped option such as sonicfill over conventional BFRC is worthwhile. The study’s null hypothesis stated that the surface texture, color stability, and the marginal discoloration tendency of sonic-activated BFRC are similar to those of conventional non-sonic activated BFRC, with no significant difference in the clinical outcomes on the basis of either the modified USPHS criteria or the FDI criteria, and to evaluate the impact on treatment choices and the reliability of both clinical criteria for evaluating resin-based composite restorations.

## Methods

### Study design

**Fig. 1:**
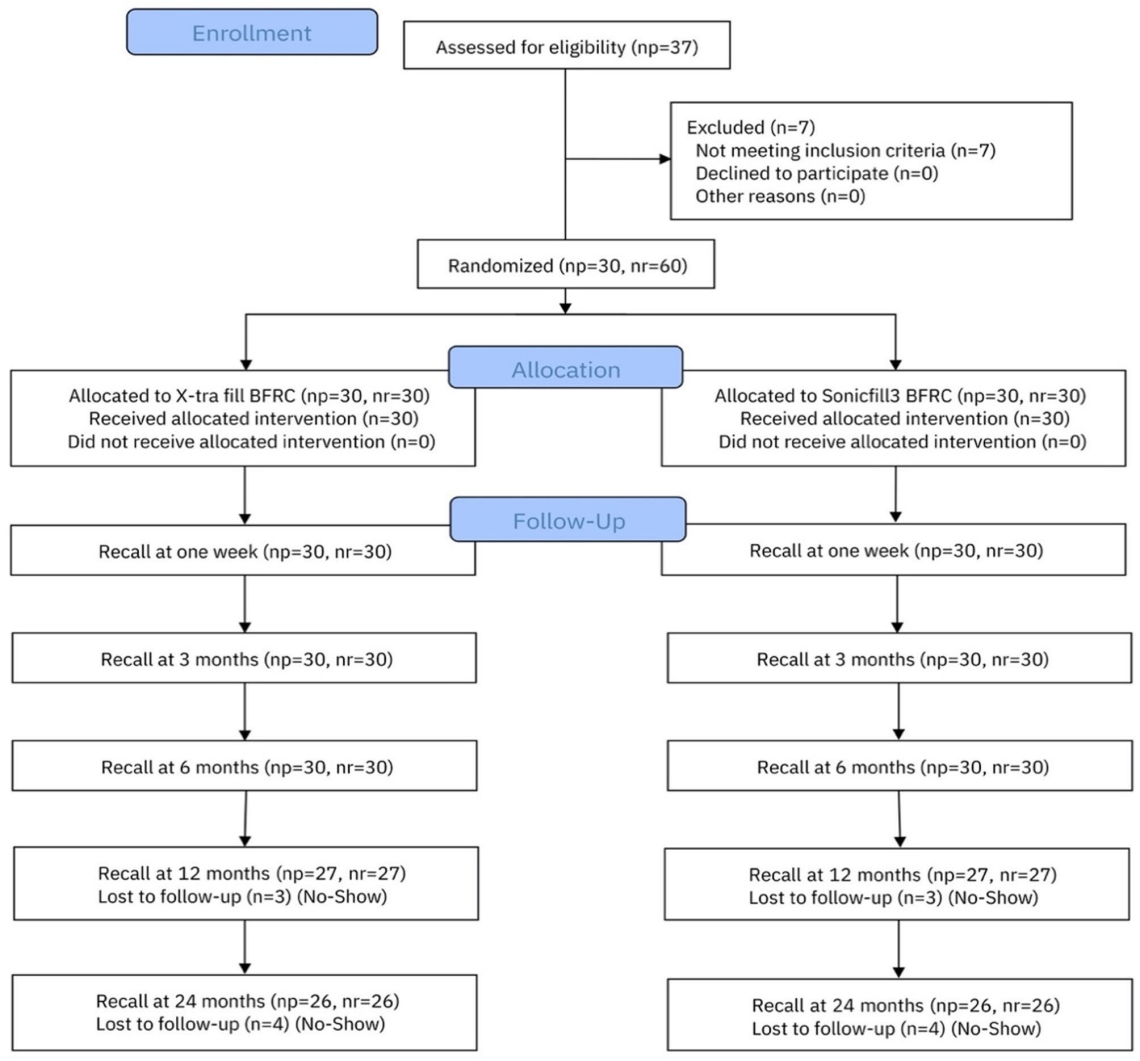
Flowchart of the current investigation (following Consolidated Standards of Reporting Trials [CONSORT] 2010)

The description of the experimental design followed the Consolidated Standards of Reporting Trials (CONSORT) statement [20] (Fig. 1). The present study was a double-blind (patients and examiners) randomized clinical trial anticipating a single-mouth design with two parallel arms, with a 1:1 allocation ratio.

### Sample size calculation

The sample size was estimated by Sealed Envelope Ltd. 2012 [21]. Considering a systematic review reported that the annual failure rate of resin composite restorations in posterior teeth was approximately 2–3% [22], the minimal sample size was 23 restorations, using an alpha of 0.05, a power of 80%, and a two-sided test to detect a difference of 15% among the test groups. Thirty teeth per group were chosen, taking into consideration the expected dropout rate over the trial period [17].

### Patient selection

Patients included in the current study sought dental treatment at the Conservative Department Clinic, Minia University. The patients were selected according to the following exclusion and inclusion criteria: aged between 18 and 45 years with good oral hygiene. A good likelihood of recall availability was included. At the same time, those who had received therapeutic irradiation to the head and neck region or had a compromised medical history, were under treatment with analgesics that might change their normal pain threshold levels, were alcoholic or heavy smokers, had participated within 6 months in a clinical trial before the beginning of this trial, and were unable to attend recall appointments were excluded.

The persons whom accepted to participate in the study had no orofacial pain or spontaneous pain, had carious lesions in their posterior teeth, either Class I or Class II cavities of comparable moderate size, with positive response to pulp testing with an electric pulp vitality tester (Model PT-20, Parkell, USA) and were detected clinically and evaluated by x-ray to note the carious lesions’ extension and to exclude the deep cavities. The chosen patients exhibited normal occlusion and had natural opposing teeth without restorations and with healthy gingival tissues, showing no indication of alveolar bone loss or recession. Additionally, patients with occlusal wear facets, clenching or heavy bruxism behaviors, temporo-mandibular joint disorders, and orthodontic therapy were excluded.

### Randomization and blinding

The randomization list was prepared by a researcher not involved in any experimental phases using a website (www.sealedenvelope.com) with an equal allocation ratio for both comparative groups. The randomization lists were individually placed in opaque, sealed envelopes after they had been numbered consecutively. Each participant was asked to pick an envelope before treatment on the day of the restorative intervention to avoid randomization pattern disclosure. The picked envelope represented the first restorative material to be used, and the tooth located in the quadrant of the higher number and more mesial in this quadrant was the first to be assigned. The material assignment was not concealed from the assistants or the main operator. Nevertheless, the recruited patients and the evaluating examiners were blinded to the allocation.

### Clinical procedure

All patients had a dental scaling and polishing session and were instructed on proper oral hygiene measures. Pre-operative photographs were taken before the restorative treatment session. Local anesthetic solution (Artinibsa 4% articaine with 1/100,000 epinephrine) was used to anesthetize each patient. Cavity preparation was performed using a T2 Racer air-driven handpiece (Sirona, Germany) after single or multiple rubber dam isolation techniques were applied according to cavity classification, using 329, 330, and 245 burs (Komet, Germany) with copious water cooling. In addition to a sharp excavator (Maillefer 57/58, Switzerland), the remaining caries were excavated with low-speed tungsten carbide burs (Komet, Germany). The exact size and form of the cavity were determined after the elimination of carious tissue, and the depth was measured with a periodontal probe (Carl Martin GmbH, Germany), where patients with very deep cavities (> 5 mm) were excluded. Yellow-coded stones (Komet, Germany) and/or hand chisels and hatchets (Nordent, USA) were used to finish the prepared cavities and remove enamel irregularities.

A separating ring (Composi-Tight 3D Fusion^™^, Garrison Dental Solutions, LLC, USA) with a suitable sectional matrix and wedge was used for matrixing. A selective etching technique was applied in the current study where enamel was etched first for 30 s using 35% phosphoric acid (HV Bisco, USA), followed by a 30 s water rinse and then drying for 5 s with moisture- and oil-free gentle airflow. Before adhesive application, cavity disinfection was performed with 2% chlorhexidine digluconate (CHX) (Consepsis, Ultradent, USA) applied for 30 s, after which the excess was removed with a dry bond brush. According to the manufacturer’s instructions, the adhesive of each manufacturer (*Futurabond M*^+^, Voco, Germany, and *OptiBond Universal*, Kerr, USA) was used and applied in a single drop with scrubbing action for an active application on both enamel and dentin for 20 s and air thinned for 5 s using oil-free airflow. A light-curing device (BluePhase N, Ivoclar Vivadent AG, Lichtenstein), fully charged, with a lamp spectrum of 385–515 nm in continuous mode that emits a light intensity of 1200 mW/cm2, was used for 20 s with direct occlusal contact. A radiometer (Blue Phase Meter II, Ivoclar Vivadent AG) inspected the curing device before each restorative procedure to ensure light irradiance.

X-tra fill RC in a compule was used to place a 4 mm increment into the prepared cavity starting from the bottom upward with the help of a titanium-coated hand instrument composite applicator. In comparison, sonicfill3 was packed using its special air-driven handpiece at a steady moderate flow-rate speed (#3) up to a 5 mm increment. After sculpting, x-tra fill RC restorations were cured for 10 s while sonicfill was cured for 20 s according to the manufacturer’s instructions, in the occlusal, buccal, and palatal/lingual directions.

**Fig. 2:**
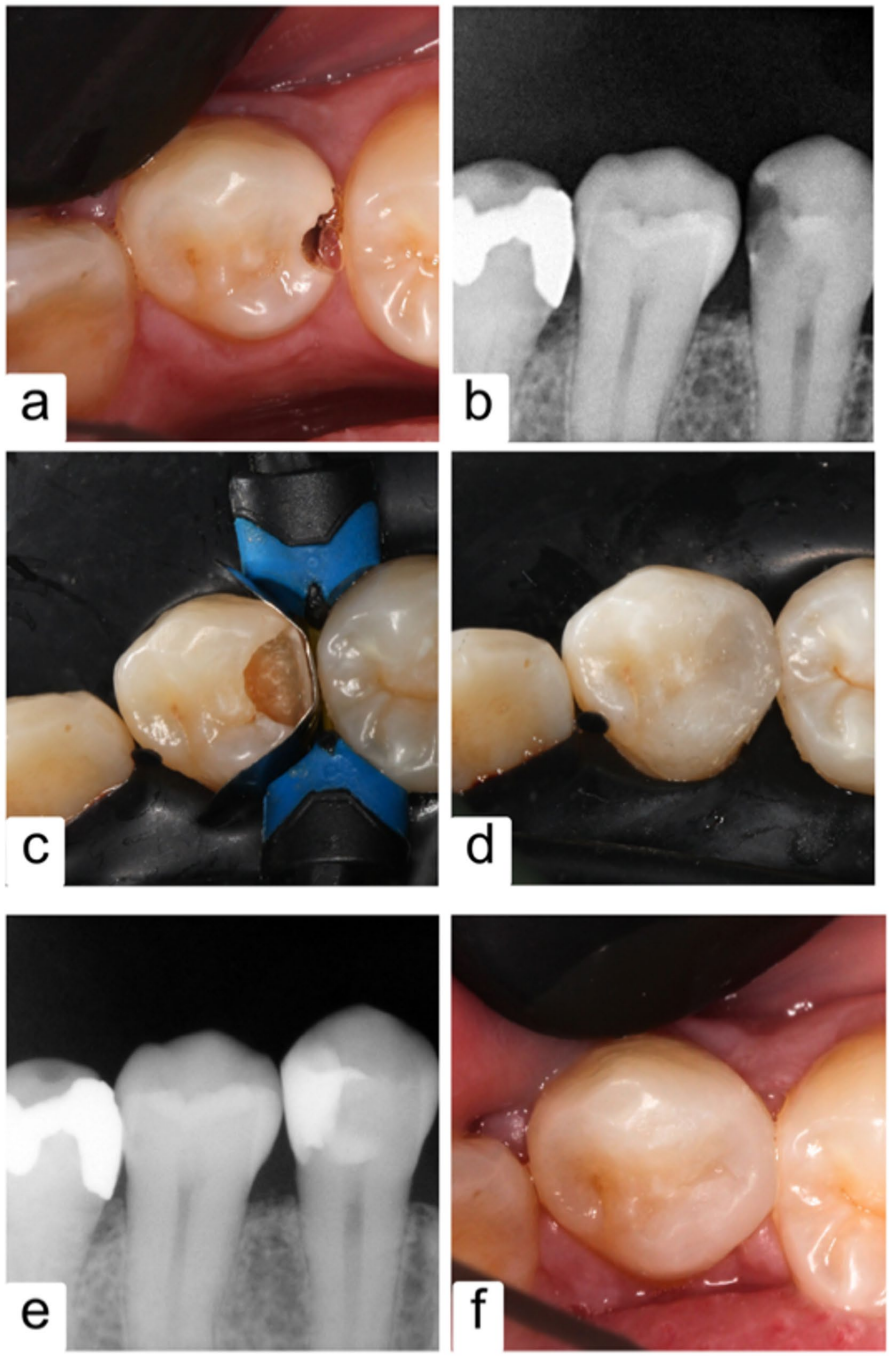
Photographs of the clinical procedures of a restoration; **a**: Preoperative clinical picture; **b**: Preoperative X-ray; **c**: Rubber dam isolation and matricing application; **d**: XF restoration immediately postoperative; **e**: Postoperative X-ray; **f**: Restoration after finishing and polishing

Using articulating paper (Bausch, Nashua, NH, USA), any premature contact was checked in all restorations and then adjusted and finished simultaneously using low-speed fine-grit diamond finishing stones (Komet, Germany) and copious water. Finally, the restorations were polished using EVE DiaComp Twist rubber polishers (EVE Ernst Vetter GmbH, Germany) and OptiShine-impregnated brush (Kerr, USA). Cavity preparation and restorations were performed with the use of 3.5x magnifying loupes. 60 cavities were prepared in a total of 30 participants. Figure 2 shows the different clinical steps of a restoration, whereas Table 1 describes the materials used for different restorations.

**Table 1:** Materials’ specifications, compositions, manufacturers, and LOT numbers

Material	Specification	Composition	Manufacturer	LOT #
***Sonicfill3***	Nano-hybrid Bulk-fill Resin Composite (Shade A2)	Bis-GMA^a^, TEGDMA^b^, Bis-EMA^c^, SiMA^d^, Glass Oxide, Silicon Dioxide	Kerr, Orange, CA, USA kerrpromo@kerrdental.com	9,226,683
***Optibond Universal***	Universal Adhesive	Acetone, Ethyl Alcohol, GPDM^e^, TEGDMA, Mineral Fillers, Ytterbium Fluoride, Photo-Initiators, Accelerators, Stabilizers, Water		9,843,748
***X-tra fill***	Micro-hybrid Bulk-fill Resin Composite (Universal Shade)	Bis-GMA, UDMA^f^, TEGDMA	Voco, Cuxhaven, Germany service@voco.de	2,148,418
***Futura*** ***bond M*** ^***+***^	Universal Adhesive	Bis-GMA, HEMA^g^, Ethanol, Water, HEDMA^h^, Methacrylate Phosphoric Acid Ester, Methacrylate Functionalized Polyacid, UDMA, Initiators, Stabilizers		2,147,450

Abbreviations: (a) bisphenol-A diglycidyl methacrylate, (b) triethylene glycol dimethacrylate, (c) bisphenol-A ethoxylated dimethacrylate, (d) silicon containing dimethacrylate, (e) glycerol phosphate dimethacrylate, (f) urethane dimethacrylate, (g)  hydroxyethyl metacrylate, (h) 1,6-exanodiol dimethacrylate

### Clinical assessment

Two calibrated, experienced, blinded examiners assessed the clinical performance of the restorations clinically following both the modified USPHS and the FDI criteria [15]. They were pre-calibrated on 5 patients not included in the study at 90% reliability, in addition to FDI calibration figures for different clinical examples [10]. Follow-up was accomplished through 1 week as a baseline [18, 19] and at 3, 6, 12, and 24 months, representing T0, T1, T2, T3, and T4, respectively.

Color assessment was made following the modified USPHS criteria for surface texture, marginal discoloration, color match, and color stability as mentioned in Table 2, whereas the aesthetic properties of BFRC restorations were also assessed using the FDI criteria, including surface luster and texture, marginal staining, and color match as stated in Table 3.

**Table 2:** Modified United States Public Health Service (USPHS) evaluation criteria

Category	Scores	Criteria
Surface texture (**ST**)	Alpha (**A**)	Smooth surface
	Bravo (**B**)	Slightly rough or pitted
	Charlie (**C**)	Surface deeply pitted, irregular grooves
	Delta (**D**)	Deeply pitted beyond repair and must be replaced
Marginal discoloration (**MD**)	Alpha (**A**)	No discoloration evident
	Bravo (**B**)	Slight staining can be polished away
	Charlie (**C**)	Obvious staining cannot be polished away
	Delta (**D**)	Staining that necessitates replacement at once
Color match (**CM**)	Alpha (**A**)	Restoration matches adjacent tooth structure in color and translucency
	Bravo (**B**)	The mismatch is within an acceptable range of tooth color and translucency
	Charlie (**C**)	The mismatch is outside the acceptable range
	Delta (**D**)	Mismatch. Restoration must be replaced immediately

Abbreviations: A, clinically ideal; B, clinically acceptable; C, clinically unacceptable, D, require immediate replacement

**Table 3:** World dental federation (FDI) evaluation criteria

Category	Scores	Criteria
Surface luster and texture (**A1**)	1	Surface luster and surface texture comparable to enamel
	2	Slightly dull surface luster and/or surface texture with minor deviations
	3	Dull surface luster and/or surface texture with multiple pores
	4	Rough surface cannot be masked by saliva film and simple polishing is not sufficient.
	5	displeasing dull surface luster and/or very rough, plaque retentive surface
Marginal staining (**A2**)	1	No discoloration evident
	2	Minor marginal staining, easily removable by polishing
	3	Moderate marginal staining but not esthetically unacceptable
	4	Pronounced marginal staining; major intervention necessary for improvement.
	5	Deep marginal staining, repair not possible
Color match and translucency (**A3**)	1	No deviation in shade, translucency/opacity with neighboring dental hard tissue. No surface staining.
	2	Minor deviation. Minor staining.
	3	Distinct deviation and/or moderate staining but not esthetically unacceptable.
	4	Localized, displeasing deviation/unacceptable surface staining, can be improved by repair.
	5	Generalized, displeasing deviation/severe surface staining, not accessible for intervention

Abbreviations: 1, clinically very good; 2, clinically good; 3, clinically satisfactory; 4, clinically unsatisfactory; 5, clinically poor

The assessment of the composite restorations’ color was clinically assessed visually using both dental and natural sources of light using eye loupes 3.5x, by the calibrated blinded assessors and further, the assessment was also done using intraoral photographs (Canon 700D with Canon macro lens 100 mm 1:2.8 USM and Viltrox macro ring lite JY670C) taken on each follow-up appointment starting from the baseline until the end of the follow-up visits. Both evaluators came to a mutual conclusion to make their final single decision in the presence of any disagreements during the evaluation.

### Statistical analysis

IBM SPSS Statistics for Windows, version 29 (IBM Corp., Armonk, NY, USA) was used for data entry and statistical analysis. The Friedman test was used to compare clinical evaluation scores across follow-up periods, while the Kruskal-Wallis test was used to compare scores among the tested groups. For pairwise comparisons, the Wilcoxon signed-rank test and Mann-Whitney U test were used for follow-up periods and between two groups, respectively. The significance level was set at *p* < 0.05.

## Results

**Fig. 3:**
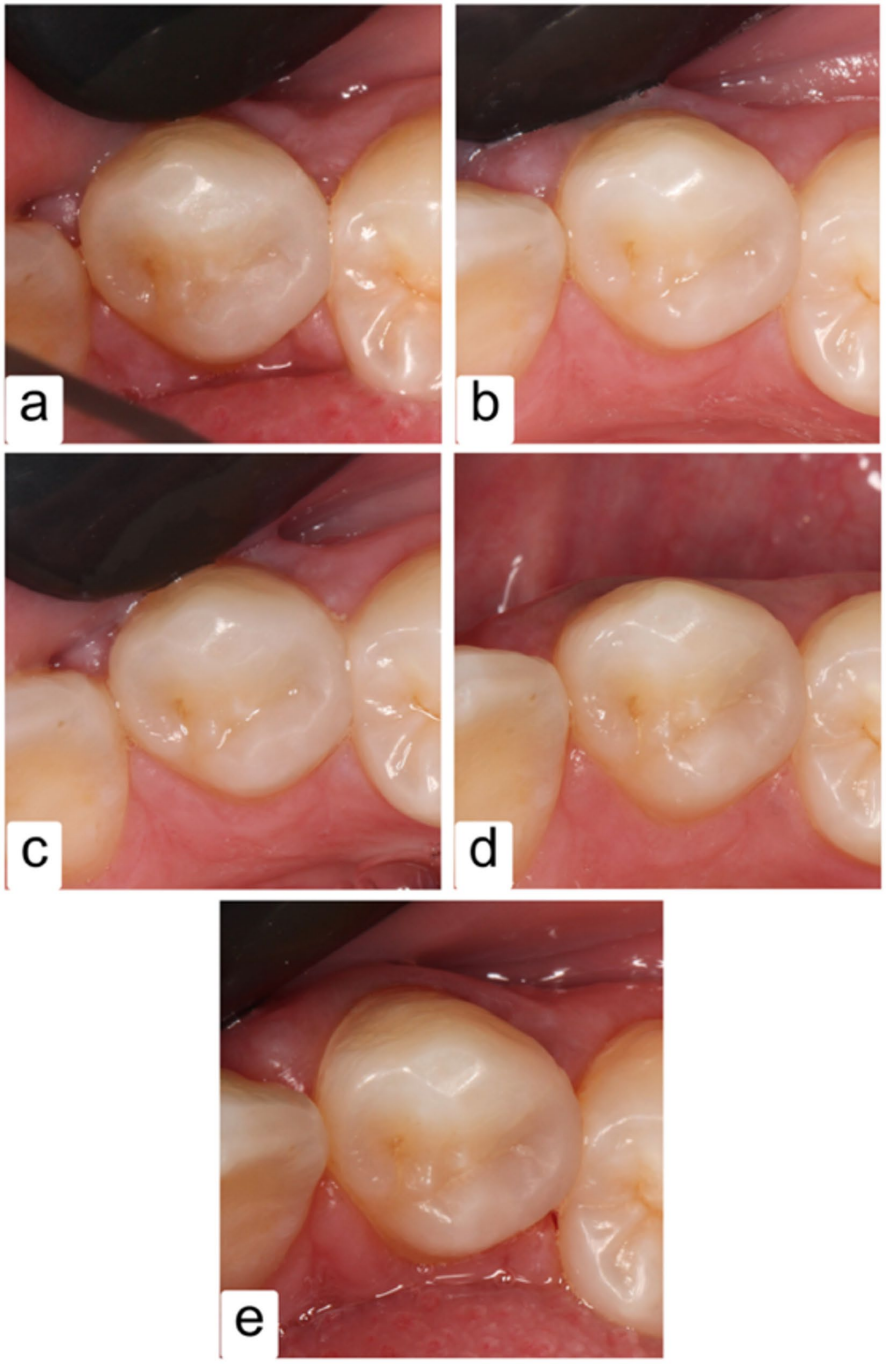
Clinical photographs of follow-up appointments of a restoration until 24 months; **a**: Baseline follow-up (1 week); **b**: 3-month follow-up; **c**: 6-month follow-up; **d**: 12-month follow-up; **e**: 24-month follow-up

Analysis of demographic data revealed a statistical insignificance relationship with all of the following aspects: age, gender, position, cavity classification, and whether the tooth restored was molar or premolar (Table 4). All the restorative procedures were conducted exactly as intended without any additional modifications and all the patients attended the follow-up appointments until 6 months. However, a few dropped out after that: 3 didn’t show up at the first-year follow-up and 4 at the second year (Fig. 3). Despite that, their absence did not affect the results statistically.

**Table 4:** Mean ± SD for independent sample t-tests and frequencies (n) for chi-square tests of demographic data between Sonicfill (SF) and x-tra fill (XF) composite groups

Demographic data			Comparison groups		*P*-value
			SF	XF	
**Sex** (n)	Male		13	13	1.000
	Female		17	17	
**Age**	Mean ± SD		34.47 ± 1.32	34.47 ± 1.32	1.000
**Tooth** (n)	Upper	Premolar	7	6	0.988
		Molar	8	8	
	Lower	Premolar	6	6	
		Molar	9	10	
**Cavity** (n)	Class I		14	15	0.796
	Class II		16	15	

### Comparison between groups

**Fig. 4:**
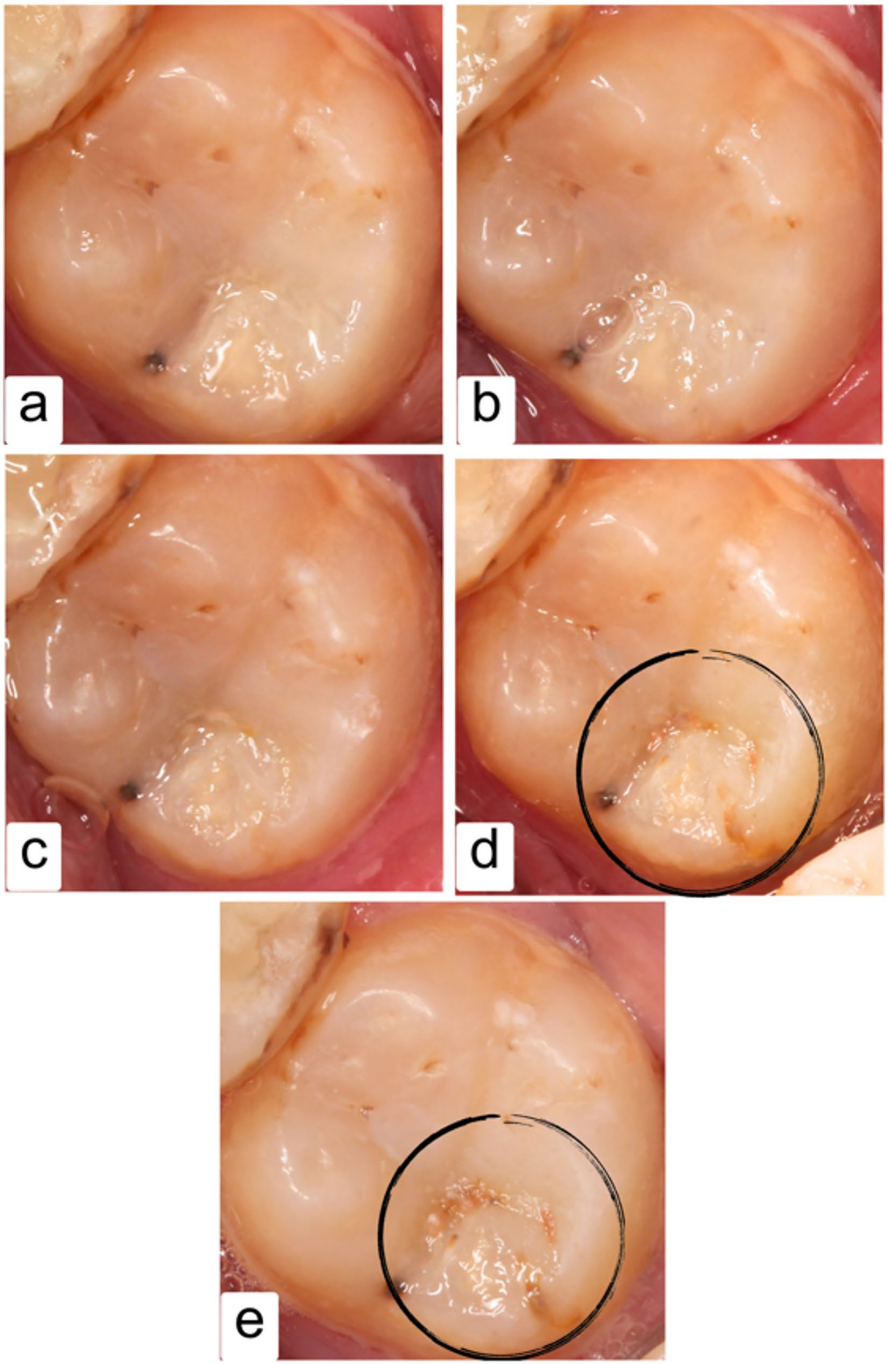
An XF restoration showed a slightly pitted surface after 12 months; **a**: Baseline follow-up (1 week); **b**: 3-month follow-up; **c**: 6-month follow-up; **d**: 12-month follow-up; **e**: 24-month follow-up

**Fig. 5:**
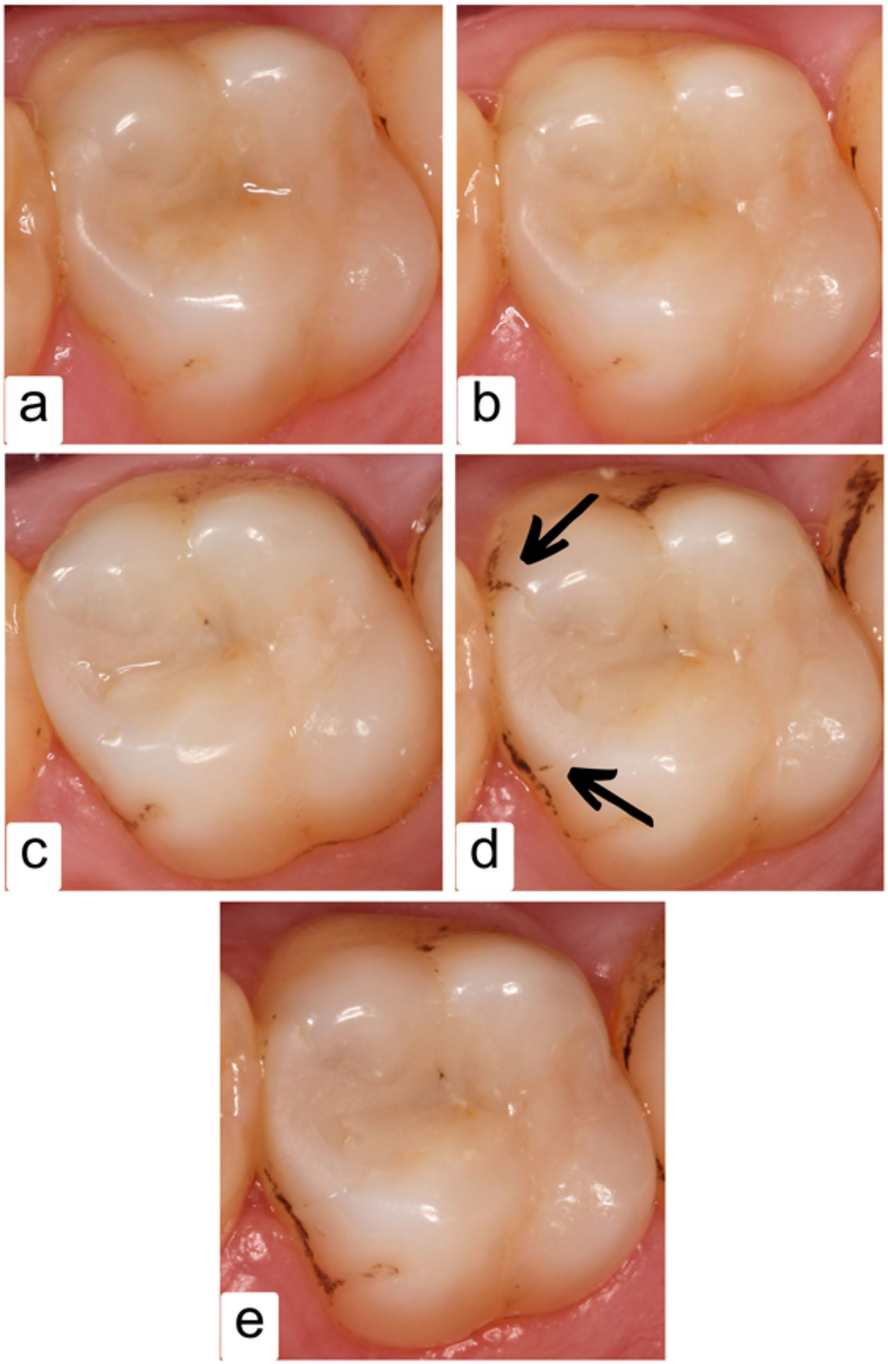
An SF restoration showed slight marginal staining after 12 months; **a**: Baseline follow-up (1 week); **b**: 3-month follow-up; **c**: 6-month follow-up; **d**: 12-month follow-up; **e**: 24-month follow-up

According to the USPHS criteria, all the restorations in the two groups revealed Alpha score till 6 months, while after 1 year, only one XF restoration showed Bravo score for surface texture (ST) (Fig. 4). On the other hand, after one year, one SF restoration showed Bravo score for marginal discoloration (MD) and two XF restorations presented two Bravo scores but there was no significant difference between the two groups (*P* > 0.05) (Fig. 5). Although that, color match (CM) scores between groups showed a borderline significant difference from the first week of evaluation (*p*-value = 0.083). Still, these differences were not statistically significant, and all restorations were considered clinically successful.

**Fig. 6:**
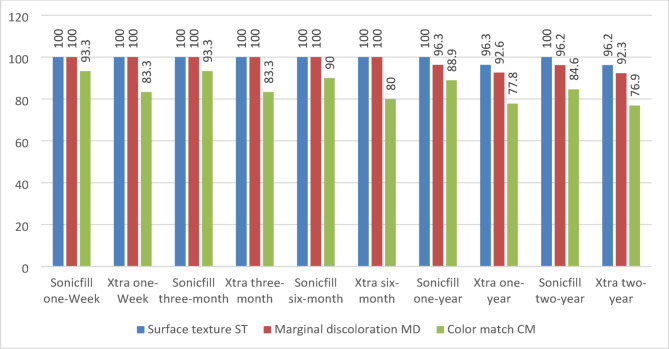
Comparison of the performance of sonicfill vs. x-tra fill restorations after two years, depending on modified USPHS criteria scores

After two years, Fig. 6 shows that the SF group had a slightly higher average score for ST (100% vs. 96.3% for XF), MD (96.2% vs. 92.3% for XF), and CM (88.9% vs. 77.8% for XF), where the score represents how many restorations have an Alpha score over the Bravo score according to the modified USPHS criteria. However, it’s important to consider that these differences were not statistically significant based on the *p*-values.

**Fig. 7:**
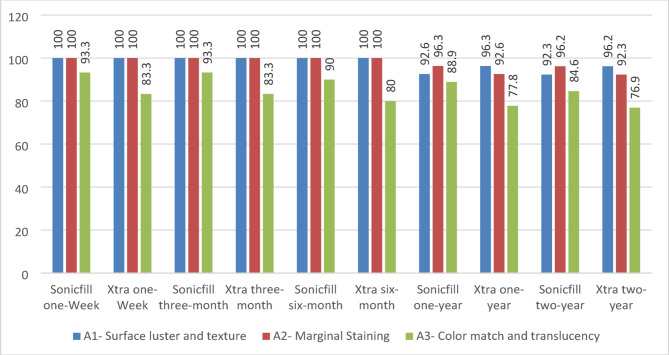
Comparison of the performance of sonicfill vs. x-tra fill restorations after two years, depending on FDI criteria scores

Regarding FDI criteria, both groups also showed satisfying clinical performance, with scores of One and Two for most restorations. However, only one restoration in the SF group and two in the XF group showed score Three after 12 months regarding marginal staining (A2), but they were also considered clinically satisfactory and successful according to the FDI criteria (Table 5; Fig. 7).

**Table 5:** Wilcoxon signed-rank test of modified USPHS and FDI clinical assessments between the SF and XF groups over the evaluation periods

Time	Material	FDI			USPHS		
		A1	A2	A3	ST	MD	CM
Week	SF	1.00	1.00	1.07	1.00	1.00	1.07
	XF	1.00	1.00	1.17	1.00	1.00	1.17
	*P*-value	1.00	1.00	0.08	1.00	1.00	0.08
3 months	SF	1.00	1.00	1.07	1.00	1.00	1.07
	XF	1.00	1.00	1.17	1.00	1.00	1.17
	*P*-value	1.00	1.00	0.08	1.00	1.00	0.08
6 months	SF	1.00	1.00	1.10	1.00	1.00	1.10
	XF	1.00	1.00	1.20	1.00	1.00	1.20
	*P*-value	1.00	1.00	0.08	1.00	1.00	0.08
12 months	SF	1.07	1.07	1.11	1.00	1.04	1.11
	XF	1.04	1.15	1.22	1.04	1.07	1.22
	*P*-value	0.32	0.56	1.00	0.32	0.32	0.32
24 months	SF	1.08	1.08	1.15	1.00	1.04	1.15
	XF	1.04	1.15	1.23	1.04	1.08	1.23
	*P*-value	1.00	0.32	1.00	0.32	0.32	1.00

### Changes within each group

In the SF group, two restorations achieved a score of Two for surface luster (A1) according to the FDI criteria after one year. Although the *p*-value of A1 was 0.09, this trend did not reach statistical significance. Whereas according to the modified USPHS criteria for surface texture (ST), none were changed even after two years (Table 6). After two years, SF restorations achieved a range of 85–96% “clinically very good” ratings on the basis of FDI criteria, whereas according to the USPHS criteria, they achieved a range of 84.6–100% “clinically ideal” ratings, but the difference was not statistically significant.

On the other hand, the XF group showed two restorations after one year, with a Bravo score according to the MD evaluation of the USPHS criteria and a score of Three according to A2 evaluation of the FDI criteria. Despite that and a Friedman test suggested a trend (*p*-value = 0.092), the difference was not statistically significant. The change over time in all other criteria of investigation of USPHS or FDI was found to be non-significant (Table 6), with all restorations considered clinically successful/sufficient.

**Table 6:** Friedman ranks of the SF and XF groups over 24 months

Material	Criteria	Assessment	Friedman Mean Rank					*P*-value
			Week	3 months	6 months	12 months	24 months	
SF	FDI	**A1**	2.92	2.92	2.92	3.12	3.12	0.09
		**A2**	2.96	2.96	2.96	3.06	3.06	0.41
		**A3**	2.92	2.92	3.02	3.02	3.12	0.23
	USPHS	**ST**	3.00	3.00	3.00	3.00	3.00	1.00
		**MD**	2.96	2.96	2.96	3.06	3.06	0.41
		**CM**	2.92	2.92	3.02	3.02	3.12	0.23
XF	FDI	**A1**	2.96	2.96	2.96	3.06	3.06	0.41
		**A2**	2.92	2.92	2.92	3.12	3.12	0.09
		**A3**	2.94	2.94	3.04	3.04	3.04	0.41
	USPHS	**ST**	2.96	2.96	2.96	3.06	3.06	0.41
		**MD**	2.92	2.92	2.92	3.12	3.12	0.09
		**CM**	2.94	2.94	3.04	3.04	3.04	0.41

## Discussion

When selecting a restorative material, color stability is considered highly important, but it could be a reason that necessitates its replacement in the future. Although multiple studies have been conducted on the effects of various beverages on RCs, little is known about the color stability of BFRC. Multiple factors, including the resin matrix-to-filler ratio and hydrophilicity of the organic matrix, in addition to differences in staining causes, can be responsible for color changes [4]. Bulk fill resin composites were chosen in this study for their specific characteristics: the fillers were modified to improve the light transmission depth, as a close correspondence of the refractive index is pursued by manufacturers between the filler and organic matrix to achieve high translucency for better conversion, whereas the resin matrix, which enables stress relief during polymerization, generally provides a crosslinked network [23]. Some physical properties affect the discoloration potential of BFRC, such as water sorption, in addition to the chemical differences in resin monomers and their concentrations, which may affect their degree of conversion. The longevity of an RC may be decreased by plasticizing and expanding its components due to water sorption, causing hydrolysis of the silane coupling agents, which allows penetration of stains [24].

Sonicfill BFRC was chosen for this study because of its ease of application and good adaptation to the cavity. Additionally, the manufacturer claims that using their special air-driven handpiece to dispense the composite while sonic vibration is applied reduces the material’s viscosity by 84%, promoting better adaptation [25]. Once the sonication is stopped, the viscosity of the material increases [26].

Compared with other methacrylate monomers, such as UDMA, the resin matrix based on Bis-GMA presents higher water uptake due to its hydrophilicity, resulting in higher staining susceptibility [27]. Water sorption can even increase more when Bis-GMA resins are combined with TEGDMA to manage viscosity, making them more susceptible to increased staining. To confirm or deny this, both tested BFRCs are Bis-GMA-based resins combined with TEGDMA.

The USPHS is the most common criterion for the clinical evaluation of restorations [15, 26]. In this study, any color change or instability of the color was ranked according to the modified USPHS criteria using visual color match evaluation in addition to dental photograph assessment [28]. However, these methods have demonstrated limited sensitivity to expose differences compared with other criteria, and the categories may not fully reflect the clinical success of the restorations. In 2007, new clinical grading criteria were approved by the Science Committee of the World Dental Federation (FDI) and the General Assembly in 2008 as “standard criteria” that should be applied when operative techniques and restorative materials are clinically investigated [10]. The FDI criteria are categorized into five scores, three of which are acceptable. Therefore, the current study was designed to also use FDI criteria in clinical evaluation to overcome such drawbacks [26], and the results may convince researchers about the criteria to be used in clinical evaluation. The FDI evaluations, compared with the USPHS criteria, were more detectable to slight changes in clinical outcomes as reported in a 12-month randomized clinical study evaluating adhesion strategies [29]. Additionally, the limited sensitivity of the USPHS was indicated by Göstemeyer et al. in entirely reflecting the restorations’ clinical success [30].

In terms of marginal compatibility and marginal discoloration, a study in which total-etch and self-etch systems were applied for 36 months reported that the FDI criteria were more sensitive than the USPHS criteria were [31]. It was also observed in the marginal discoloration assessment by Begec and Bahsi that the FDI criteria provided more sensitive results [15]. For the FDI criteria, scores of 1 and 2 represent the main difference from the USPHS criteria, which are subtle and more prone to disagreements, whereas the results related to the Alpha and Bravo scores of the USPHS criteria are less subjective [32]. A published review by Marquillier et al. [33] highlighted the increasing adoption of the FDI criteria in clinical trials, with usage increasing from 4.5% in 2010 to 50.0% in 2016. The current FDI criteria have been enhanced through a structured process to gather information from a panel of experts through a series of meetings and/or evaluations [14].

According to both the USPHS and FDI criteria, 100% of the restorations in this research were considered clinically successful/sufficient, as the restorations had either Alpha or Bravo scores with good clinical performance according to the USPHS criteria or were clinically accepted with scores of 1, 2, or 3 scores according to the FDI criteria. Therefore, no statistical comparison was performed. However, another study [32] assessed the inter-examiner reliability and the reproducibility of both clinical criteria for evaluating restorations. They reported that for both criteria, inter-examiner agreement was excellent for treatment decisions with no difference between criteria found regarding clinical decision-making, irrespective of the examiner.

The surface texture, marginal discoloration, and color match of both restorations lie in Alpha and Bravo only according to the modified USPHS criteria and 1 and 2 according to the FDI criteria. While both tested BFRCs are Bis-GMA resins combined with TEGDMA which were previously documented as being more susceptible to increased staining, the results revealed that there was no change in the tested color parameters, with a non-significant difference in the tested items throughout the testing periods. Similarly, statistically, there were no significant differences in the clinical performance regarding aesthetics between the two BF materials over 2 years. On the basis of both evaluation criteria, the null hypothesis was supported by the results of the present study and accepted.

However, SF showed better scoring numbers according to evaluation criteria, either USPH or FDI, which match the reported scores of other studies [8, 18, 34, 35]. This outcome also coincides with various studies where SF showed the highest color stability with respect to XF and other BFRC materials subjected to a staining broth composed of orange juice, grape juice, strawberry fruit punch, coffee, and tea [36]. Akalin et al. also reported that the color stability of SF was acceptable after 2 years, which may be related to the filler morphology and resin matrix [9]. SF, which includes Bis-EMA as a highly hydrophobic monomer, compared with a conventional Bis-GMA RC (Nova Comp-N, Imicryl, Konya, Turkey), has higher color stability [37]. SF also contains a lower polymerization shrinkage monomer, i.e., the SiMA monomer, which is characterized by lower solubility, lower water sorption, and higher flexural strength than Bis-GMA RCs [38]. In addition, it showed the highest color stability among all the investigated BFRCs for most of the staining liquids [36, 39].

Moreover, others reported that XF, as a micro-hybrid RC, presented lower staining resistance than did SF, as a nano-hybrid RC. This finding could probably be attributed to Bis-EMA, which has lower water sorption than UDMA and is highly hydrophobic [36]. On the other hand, it was reported that the presence of TEGDMA in the SF material composition was a reason for the greater discoloration of SF than other compared RCs, whereas UDMA was found in other studied materials: a nanohybrid RC (Nova Compo N, Imicryl, Konya, Turkey) and a compomer (Dyract-XP, Dentsply, DeTrey, Konstanz, Germany) [40].

The greater probability of failure and gradual deterioration of the adhesive interface may be related to marginal discoloration over time. Atabek et al. [3] compared incremental RC to sonic-activated BFRC; they reported that the variable marginal discoloration after 24-month of follow-up had the same scores. The hydrolytic degradation anticipated in self-etching adhesives was the cause of these outcomes, rather than the difference between the techniques used and the two types of RCs, similarly to another two studies that compared bulk-fill resins in single-increment, either sonicated or not, with incremental RCs [8, 19]. Marginal discoloration scored 100% Alpha in the final SF evaluation as reported by Bayraktar et al. in their study [18]. In comparison, restorations by incremental RC and single-filling consistency resins of other groups presented a 2.33% Bravo score. They also reported that the success of their results may be related to the sonic energy applied to the composite resins of the intervention group and the use of the manufacturer’s suggested adhesive for each restorative material.

The bond longevity of all-in-one adhesives is ultimately affected by their extreme hydrolytic breakdown, as reported by Atabek et al. [3], which could explain the changes in marginal discoloration over time. Bayraktar et al. [18] reported similar results. After one year, they observed a very minor degree of marginal discoloration in relation to the adhesive degradation over time in both the bulk-fill and the conventional RCs. Colored molecules infiltrating the interface and/or inside the adhesive layer may cause marginal staining [41].

Additionally, the tendency for discoloration can be affected by the surface texture of RC materials, which also affects plaque accumulation, increasing the risk of gingival inflammation and secondary caries [36]. Therefore, the current study used a finishing and polishing protocol for recontouring and removing overhangs. Karadaş and Demirbuğa measured surface roughness (Ra) in their study, and XF attained the highest values. This might be attributed to having the largest fillers according to their micro-hybrid nature. On the other hand, the surfaces of the other tested RCs were similar to those of the other nanocomposites, including SF. However, no significant differences were found when compared to each other [36].

Dental photography played an important role during evaluation in this research as an adjunct restoration assessment tool. Larasati et al. [13], in their study in which both criteria, modified USPHS and FDI, were used, reported the efficacy of digital image-based assessments for glass ionomer cement restorations in primary teeth. This finding is important for future studies and for guiding dental practitioners by providing insights into appropriate evaluation criteria for reliable digital image-based assessments of restorations, especially those related to occlusal restorations.

One of the main limitations of the present study is the relatively short follow-up period (24 months). Although long-term follow-up studies can provide more information than short-term follow-up studies, it can also be quite challenging to maintain a group of participants’ attendance for a long time, and we can still learn some valuable information regarding the clinical performance of materials from short-term clinical trials [1]. Nevertheless, further long-term clinical studies are needed. Other limitations are related to the potential for operator bias that hasn’t been blinded because of using different tools and application processes for each BFRC. Also, the limited shade option of x-tra fill BFRC (universal shade) might affect the evaluation of color match criteria in comparison to the A2 shade of sonicfill BFRC, which might be more natural-mimicking.

## Conclusions

It could be concluded after 24 months, under the limitations of this study, that there was no significant difference observed regarding the surface texture, color stability, and marginal discoloration of both bulk-fill resin composites, the sonicfill and x-tra fil, and both were considered clinically successful. However, long-term clinical trials are still needed for a better assessment of clinical performance.

Both the modified USPHS criteria and the FDI criteria were reliable and comparable, to standardize the evaluation of the aesthetic properties of the bulk-fill resin composites included in this study, with no significant difference between the two criteria.

### Clinical relevance

Dentist’s treatment decisions would be guided by the ability to assess the clinical performance of different restorative materials or techniques according to different evaluation criteria. In addition, the ease of handling and application of sonic-activated over non-sonic-activated BFRC when other criteria are similar could be considered.

## Data Availability

The datasets used and/or analyzed during the current study are available from the corresponding author upon reasonable request.

## Abbreviations

A1: Surface Luster
A2: Marginal Staining
A3: Color Match and Translucency
BFRC: Bulk-fill Resin Composite
Bis-EMA: Bisphenol-A Ethoxylated Dimethacrylate
Bis-GMA: Bisphenol-A Diglycidyl Methacrylate
CHX: Chlorhexidine Digluconate
CM: Color Match
CONSORT: Consolidated Standards of Reporting Trials
FDI: World Dental Federation
GPDM: Glycerol Phosphate Dimethacrylate
HEDMA: 1,6-Exanodiol Dimethacrylate
HEMA: Hydroxyethyl Metacrylate
MD: Marginal Discoloration
RC: Resin Composite
SF: Sonicfill
SiMA: Silicon Containing Dimethacrylate
ST: Surface Texture
TEGDMA: Triethylene Glycol Dimethacrylate
UDMA: Urethane Dimethacrylate
USPHS: United States Public Health Services
XF: X-tra Fill
